# Live virus-free or die: coupling of antivirus immunity and programmed suicide or dormancy in prokaryotes

**DOI:** 10.1186/1745-6150-7-40

**Published:** 2012-11-14

**Authors:** Kira S Makarova, Vivek Anantharaman, L Aravind, Eugene V Koonin

**Affiliations:** 1 National Center for Biotechnology Information, National Library of Medicine, Bethesda, MD, 20894, USA

## Abstract

**Background:**

The virus-host arms race is a major theater for evolutionary innovation. Archaea and bacteria have evolved diverse, elaborate antivirus defense systems that function on two general principles: i) immune systems that discriminate self DNA from nonself DNA and specifically destroy the foreign, in particular viral, genomes, whereas the host genome is protected, or ii) programmed cell suicide or dormancy induced by infection.

**Presentation of the hypothesis:**

Almost all genomic loci encoding immunity systems such as CRISPR-Cas, restriction-modification and DNA phosphorothioation also encompass suicide genes, in particular those encoding known and predicted toxin nucleases, which do not appear to be directly involved in immunity. In contrast, the immunity systems do not appear to encode antitoxins found in typical toxin-antitoxin systems. This raises the possibility that components of the immunity system themselves act as reversible inhibitors of the associated toxin proteins or domains as has been demonstrated for the *Escherichia coli* anticodon nuclease PrrC that interacts with the PrrI restriction-modification system. We hypothesize that coupling of diverse immunity and suicide/dormancy systems in prokaryotes evolved under selective pressure to provide robustness to the antivirus response. We further propose that the involvement of suicide/dormancy systems in the coupled antivirus response could take two distinct forms:

1) induction of a dormancy-like state in the infected cell to ‘buy time’ for activation of adaptive immunity; 2) suicide or dormancy as the final recourse to prevent viral spread triggered by the failure of immunity.

**Testing the hypothesis:**

This hypothesis entails many experimentally testable predictions. Specifically, we predict that Cas2 protein present in all *cas* operons is a mRNA-cleaving nuclease (interferase) that might be activated at an early stage of virus infection to enable incorporation of virus-specific spacers into the CRISPR locus or to trigger cell suicide when the immune function of CRISPR-Cas systems fails. Similarly, toxin-like activity is predicted for components of numerous other defense loci.

**Implications of the hypothesis:**

The hypothesis implies that antivirus response in prokaryotes involves key decision-making steps at which the cell chooses the path to follow by sensing the course of virus infection.

**Reviewers:**

This article was reviewed by Arcady Mushegian, Etienne Joly and Nick Grishin. For complete reviews, go to the Reviewers’ reports section.

## Background

**Figure 1:**
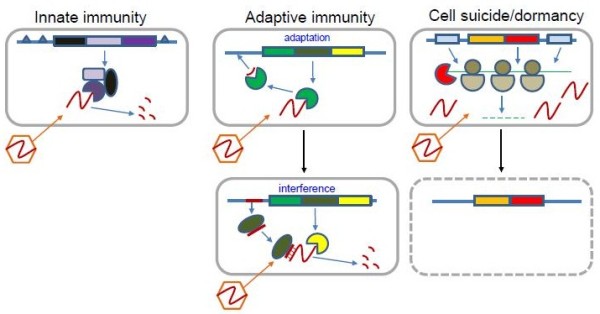
The defense systems in prokaryotes: innate immunity, adaptive immunity and programmed suicide/dormancy.

Viruses are the most abundant biological entities on earth. In well-characterized habitats such as seawater and soil the number of viral particles exceeds the number of cells by one to two orders of magnitude [1-3]. Thus, all bacteria and archaea exist in a perennial arms race with the excessively abundant viruses [4,5]. Consequently, prokaryotes have evolved extremely diverse and elaborate antiviral defense systems that occupy a substantial part of the genome in all free-living prokaryotes [6]. Antivirus defense systems can be classified into two broad categories that differ in their general principles of action. Immune systems function on the self-nonself discrimination principle, i.e. specifically recognize and destroy foreign genomes while protecting the host genome (Figure 1). In addition to targeting viral genomes, these systems are also involved in other inter-genomic conflicts with selfish elements such as plasmids. In contrast, suicide systems execute cell death or dormancy programs that prevent a virus from completing its reproduction in the given infected cell and subsequently infecting other cells [7]. The self-nonself discrimination principle is employed, in particular, by the restriction-modification (RM) systems, which are probably the best characterized defense systems in prokaryotes, to a large extent, because restriction endonucleases are essential experimental tools of molecular biology [8-10]. Recently, an analogous system of DNA phosphorothioation (known as the DND system) has been characterized [11-13]. The RM and DND systems may be considered mechanisms of innate immunity: they do not adapt to a specific infectious agent but simply ensure protection of the self DNA and attack non-self invaders indiscriminately. The CRISPR (clustered regularly interspaced short palindromic repeats)-Cas (CRISPR-associated genes) systems that are encoded in the genomes of the great majority of archaea and many bacteria represent another type of defense machinery that is also based on self-nonself discrimination but fits the definition of adaptive (as opposed to innate) immunity [14-18]. Unlike the RM and DND systems that generically distinguish between modified and unmodified recognition sites in DNA, the CRISPR-Cas systems function via adaptation to a specific infectious agent. The CRISPR-Cas first incorporates a unique fragment of the invading DNA into a specific locus in the host genome and then employs the transcripts of these unique spacers to target the cognate sequences in viral or plasmid genomes, achieving extremely high levels of immunity as the result. 

The toxin-antitoxin (TA) and abortive infection (ABI) systems that are nearly ubiquitous and highly abundant in bacteria and archaea typically cause cell suicide or dormancy in response to virus infection or other forms of stress [7,19-26]. Under normal conditions, the toxin component of a TA system is kept inactive through the association with the antitoxin but under stress the antitoxin is inactivated and the toxin is unleashed. These systems employ several molecular mechanisms, the most common one being the cleavage of translated mRNA directly on the ribosomes by toxins that possess nuclease (interferase) activity. The evolution of a suicidal response via toxin action might be explained by invoking altruism/kin/group selection: when immune systems fail to prevent virus reproduction, the TA and ABI systems kill the infected cell or render it dormant, hence increasing the survival chance for the surrounding cells [7,19-26]. Such altruistic behavior is particularly likely to be selected for in situations where the neighboring cells are kin emerging from clonal expansion and/or when cells have other factors favoring cooperation, such as aggregation in a biofilm. Indeed, a recent study combining experimental analysis of virus-host interaction with mathematical modeling and simulation has shown that bacterial “Suicidal Defense Against Infection” is efficient in a spatially structured habitat but under well-mixed conditions [27]. The dormancy route not only attains the same end but additionally provides potential for survival of the infected cell itself.

Comparative genomic analysis of the genes encoding defense systems in archaea and bacteria revealed statistically significant genomic clustering of RM, ABI and TA systems (but not CRISPR-Cas) within defense islands [6]. Furthermore, several specific, tight associations were observed. In particular, the abortive infection system AbiU1/AIPR is frequently associated with RM [28]. Two additional architectural associations of the same type have been reported for two previously uncharacterized domains Ymh (Pfam09509) and DUF4145 (Pfam13643), with the latter domain being fused to restriction subunits of RM type I systems.

Whether the connection between immune and suicidal defense systems involves any form(s) of specific functional cooperation or occurs fortuitously due to non-adaptive clustering of horizontally transferred genes remains unclear [6]. However, in one well-studied model such cooperation has been discovered. This case involves the *Escherichia coli* anticodon nuclease (ACNase) PrrC which contributes to the T4 phage exclusion mechanism as a component of the RM type Ic system PrrI [29]. The *prrC* gene co-localizes with the cluster of genes for the 3 subunits of a RM system (*hsdMSR*), and this genomic association is conserved in diverse bacteria, suggestive of functional coupling. Normally, PrrC persists as an inactive, latent endoribonuclease in association with the Hsd complex but it can be allosterically activated either by unmodified DNA, or by increased levels of dTTP or by the small anti-restriction peptide encoded by the T4-like enterobacteriophages. The activated PrrC ACNase cleaves the anticodon of tRNA^Lys^ in a GTP-dependent manner, with GTP hydrolysis catalyzed by the N-terminal ABC NTPase domain of PrrC. The cleavage of tRNA^Lys^ inhibits translation and abrogates the growth of *pnk* (polynucleotide kinase) or *rnl1* (RNA ligase) mutants of T4. In wild type T4 and related phages, Pnl and Rnl1 jointly function as an antidote to PrrC by repairing the cleaved tRNA at the 2′,3′ cyclic phosphate and 5′ OH [30,31]. The ACNase RloC that is homologous to PrrC, although usually not linked directly to RM systems, seems to function under the same logic, i.e. as an antiviral contingency that acts when DNA restriction is alleviated under genotoxic stress [32,33]. Although PrrC and RloC are not known to cause bacterial suicide or persistence, they clearly function on the exact same principle, namely inhibiting translation to prevent virus reproduction in situations when an innate anti-phage immunity mechanism fails. Indeed, an analogous but completely independent mechanism of translation inhibition has been demonstrated to be critical in a parallel defense system that is deployed against T4 by certain strains of *Escherichia coli*. Here, a defective prophage produces a toxin known as Lit that contains a zincin-like metallopeptidase domain and cleaves the elongation factor Tu thereby instigating cell suicide or at least abolishing translation in the bacterial cell infected by T4. This system has been shown to prevent the spread of T4 to the remaining cells in the colony [34,35].

We were interested in the possibility that echeloning of suicidal/persistence-based defenses and directed attack on viral nucleic acids via genomic coupling could be a general phenomenon in prokaryotes. Here we present evidence of association of TA/ABI systems with RM, DND and CRISPR-Cas systems and outline the hypothesis that all classes of prokaryotic immunity systems function in direct cooperation with suicide or persistence (dormancy) mechanisms.

## Presentation of the hypothesis

**Figure 2:**
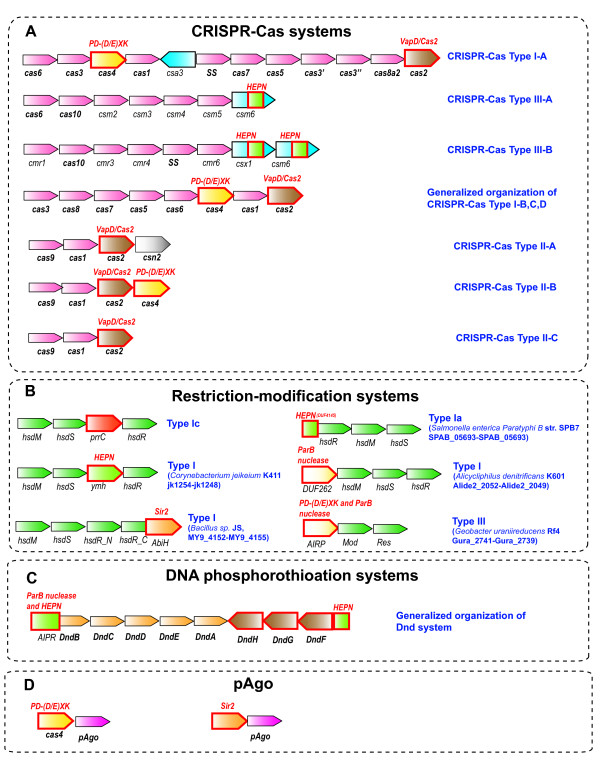
**Organization of the genomic loci that encode prokaryotic immune systems including toxin genes.** The core genes of CRISPR-Cas, RM, and DND systems in predicted operons are shown by pink arrows; genes with (predicted) toxin activity are shown by different colors, and the (predicted) toxin domains are denoted with red outline. The Csa3 protein in the Type IA system lacks the HEPN domain. Abbreviations: HEPN - higher eukarytoes and prokaryotes nucleotide-binding domain; Sir2, ParB and REase, DEDD are nucleases from distinct superfamilies. **A**. CRISPR-Cas. Gene names follow the nomenclature and classification from [18]. **B**. Restriction-modification. Gene names follow the nomenclature and classification from [46]. **C**. Phosphorothioation. Gene names follow the nomenclature from [12]. **D**. Prokaryotic argonaut, pAgo.

We first consider the CRISPR-Cas systems of adaptive immunity. The recent experimental breakthroughs seem to have revealed all the genes that are responsible for DNA/RNA targeting [15,36-43] and spacer integration [44] by CRISPR-Cas. However, several genes that are stably associated with CRISPR-Cas loci still do not fit into this scheme [45] (Figure 2A). 

The only two genes that are universal among the CRISPR-cas systems are *cas1* and *cas2* both of which are implicated in the first stage of CRISPR-Cas function, adaptation, or spacer integration [44]. However, despite the genetic evidence of the involvement of both genes, all the enzymatic activities required for adaptation appear to be provided by Cas1 alone [44]. The Cas2 protein has been shown to possess a sequence-specific endoribonuclease activity [47] and is a homolog of the VapDHi toxin of the VapDHi/VapX TA system from *Haemophilus influenzae*[47-50]. Therefore it appears highly likely that like VapDHi, Cas2 is an mRNA interferase that specifically cleaves ribosome-associated mRNAs, an activity that is mechanistically irrelevant for the adaptation stage of the CRISPR-Cas function. Furthermore, certain Cas2 homologs occur independently of an antitoxin in predicted operons or in genomic contexts corresponding to mobile selfish elements that are able to mediate their replication in conjunction with transfer. In these cases, the Cas2-homologs are typically in the neighborhood of a gene encoding a resolvase (e.g. gi: 57504998, from *Campylobacter coli* RM2228) or a Mob-type relaxase (gi: 313144877 from *Helicobacter cinaedi*). Conceivably, the endoRNase activity of these Cas2 homologs might foster addiction to the respective mobile elements via toxin-like action upon disruption of the selfish element. Furthermore, the combination of the Cas2-like protein with a DNA resolving/cleaving enzyme is reminiscent of the CRISPR-Cas systems where Cas1, the partner of Cas2, plays the key role in the integration of the acquired spacer DNA. This parallel seems to support the potential origin of the Cas1-Cas2 dyad of the CRISPR-Cas systems from an ancient mobile element [47] similar to the aforementioned elements that combine Cas2-like genes with genes encoding DNA resolving/cleaving enzymes. Therefore, we hypothesize that Cas2 retains its ancestral toxin-like EndoRNase function within the CRISPR-Cas systems but the interferase activity is kept in check through reversible inhibition by the Cas2-Cas1 interaction. This scheme is proposed as a direct analogy to the control mechanism of PrrC which is kept in its latent state via the interaction with the associated RM system.

**Figure 3:**
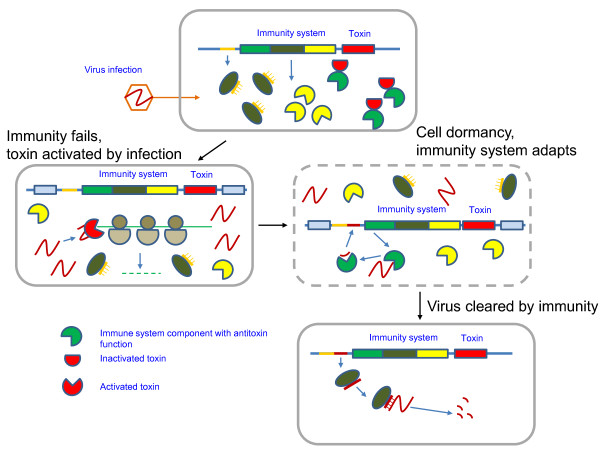
**The immunity-dormancy/suicide coupling hypothesis: Route 1 – toxin action before immunity activation.** The coupling between dormancy-suicide and immunity is specifically illustrated by the CRISPR-Cas system that is hypothesized to adapt by inserting a cognate spacer during a dormancy-like phase induced by the action of the toxin (typically, Cas2).

Under this hypothesis, when CRISPR-Cas fails to contain virus growth due to viral counter-attack and/or the level of genotoxic stress increases due to the accumulation of nucleotide metabolites synthesized by viral enzymes, Cas2 is activated (possibly through degradation of Cas1) and abrogates translation, probably leading to cell suicide or dormancy (Figure 3). The requirement of Cas2 for spacer integration might stem from regulation or stabilization of Cas1 via Cas1-Cas2 complex formation that also reversibly inactivates Cas2. Perhaps even more important, Cas2 might act at the initial stage of the CRISPR-Cas response by rendering the infected cell dormant and hence ‘buying time’ to allow the CRISPR-Cas system to integrate virus-specific spacers for effective future use in antivirus response. The strong sequence conservation of Cas2 across the CRISPR-Cas systems, together with its sequence-specific endoribonuclease activity, appear to be better consistent with “self” RNAs being targeted rather than non-self RNAs which would likely select for greater diversity of Cas2 [51]. Of interest in this regard is the fusion of Cas2 with a 3′-5′ exonuclease RNaseH fold domain (e.g. LSEI_0356 from *Lactobacillus casei* ATCC 334), which is present in several Type I-E CRISPR-Cas loci. In these fusion proteins, Cas2 appears to be inactivated as a result of substitution of the catalytic residues so that the ribonuclease activity is most likely supplied by the 3′-5′exonuclease-like domain [52]. 

Most of the Type I and Type III CRISPR-Cas systems encompass additional domains, besides Cas2, which could potentially function similarly to toxins (Figure 2A). Typically, these predicted toxins are either fused to or encoded in the same operon with proteins of COG1517 (also known as Csm6 and Csx1) that are common in CRISPR-Cas systems (Figure 2A). Most COG1517 proteins contain a distinct Rossmann-fold domain [5,49] but many in addition contain a third, usually C-terminal domain. The domains fused to COG1517 proteins include REase fold DNases (Pfam Clan: PD-(DE)xK) [49], as confirmed by a recently solved crystal structure (pdb code 1XMX), and HEPN domains ([53] and (KSM, VA, EVK, LA, unpublished). The HEPN domain has been predicted to comprise a component of a distinct TA jointly with the minimal nucleotidyltransferase domain [25]; recently, HEPN has been shown to function as a toxin [54]. Our recent analysis suggests that the PrrC/RloC ACNase domain and the KEN domain of eukaryotic unfolded protein response/ antiviral RNAses (Ire1/RNAse L) also are distinct versions of the HEPN fold and accordingly that several HEPN domains are RNases with a toxin-type activity (KSM, VA, EVK, LA, unpublished). Additional notable architectures of this CRISPR-CAS associated Rossmann-like domain include fusions to distinct RNase domains with potential toxin activity such as the RelE (e.g. sll7062 from *Synechocystis* sp. PCC 6803) and PIN (e.g. APE_2119.1, *Aeropyrum pernix* K1) [55]. Both these toxins have been shown to possess mRNA interferase activity [24]. Most of the predicted toxin-like genes are located within or in a close proximity of predicted Type III CRISPR-Cas operons [18].

Moreover, the widespread Cas4 protein, a REase fold nuclease that is part of most Type I CRISPR-Cas systems but for which no specific function in the CRISPR-Cas response so far has been established also might exhibit a toxin-like activity. In this respect three observations seem to be relevant: first, the aforementioned fusion of the REase fold nuclease domain with the Rossmannoid domain; second, the only CRISPR-Cas Type I-A locus so far detected in which the COG1517 protein (Csa3) does not contain a potential toxin domain encompasses Cas4 (KSM, unpublished); third, the fusion of Cas4 with Cas1 [18]. The Cas4-Cas1 fusion may be considered in parallel with the fusion of Cas1 with the reverse transcriptase (RT) domain that was described previously [49] and now appears to be widespread in a variety of bacteria (e.g. alr1468 from *Nostoc sp*. PCC 7120, Franean1_1369 from *Frankia sp*. EAN1pec, VVA1544 from *Vibrio vulnificus* YJ016, etc.). The RT domain fused with Cas1 is related to the RT-like proteins of the AbiA and AbiK families that participate in the abortive infection response [56]. Recently, it has been shown that AbiK is not a typical RT but apparently catalyzes non-templated synthesis of random sequence DNA that remains covalently attached to the protein and contributes to abortive infection via a still uncharacterized mechanism [57]. We propose that the Cas1-associated RT functions in a similar fashion. Thus, within the CRISPR-Cas systems, multiple cases of replacement of one type of toxin by another seem to occur.

Similarly to CRISPR-Cas, defense systems that are best classified as innate immunity are also associated with suicide/dormancy genes. In addition to the PrrC-PrrI link discussed above, many other Type I and Type III RM systems encompass domains with potential toxin-like roles including different families of HEPN domains as well as Sir2, ParB and REase fold nucleases that are homologous to abortive infection system subunits and predicted toxins of TA systems [6] (Figure 2B). Thus, in the RM system the same phenomenon of repeated toxin displacement as in the CRISPR-Cas systems seems to take place.

Although the specific functions of the proteins involved in DNA phosphorothioation and those that are involved in the restriction by this system are poorly understood, the sets of genes involved in both processes are relatively well-defined [11-13] (Figure 2C). One of the genes (*dndB*) that is part of the phosphorothioation operon is a negative regulator of the process and might play a role in discriminating between the modification of self and non-self DNA [13,58]. The N-terminal domain of DndB belongs to the AbiU1/AIPR family of abortive infection proteins that are also linked to Type III RM systems [28]. The AIPR proteins contain an N-terminal HEPN domain that is a toxin and a predicted RNase (see above). Another HEPN domain is present in the DndF protein that is implicated in the restriction process [11-13].

Other, poorly characterized prokaryotic defense systems also might contain toxin domains (Figure 2D). In particular, it has been predicted that prokaryotic Argonaut protein (pAgo) is a key component of a distinct defense system that can be encoded by a stand-alone gene or within putative operons together with several nucleases [59]. Notably, one of these nucleases is a close homolog of Cas4 that might function as a toxin in conjunction with CRISPR-Cas systems [59]. In addition, other REase fold DNases as well as a predicted Sir2 superfamily enzyme are often found in association with pAgo [59]. Thus, notably, (predicted) Sir2 superfamily enzymes are distinct components of the ABI system AbiH, and are linked both to Type I RM systems and to the putative defense system centered around pAgo (Figure 2A and 2D). These enzymes might either function as mono-ADP-ribosylating toxins targeting proteins or DNA [60] or as nucleases as has been proposed for the Sir2 enzymes found in systems containing HerA-FtsK-like proteins [61].

Most likely, there are additional, still uncharacterized innate and/or adaptive immunity defense systems in prokaryotes that are also coupled with toxins. One recently studied example is the phage and chemical stress resistance systems centered on the *ter* genes [62]. Several operons of these systems combine genes encoding nucleases and helicases similar to those found in RM systems with genes encoding COG1517 family proteins as well as putative toxins related to those in typical TA systems. The latter proteins include Doc domains, which modify proteins by addition of NMP moieties, and predicted RNases homologous to RelE, barnase or the PIN domain. It seems likely that these systems also engender higher level functional cooperation between the restriction DNAses that directly target invading DNA and toxins that promote dormancy or suicide. These operons also share components with the Pgl system of reverse restriction-modification that encompasses many genes whose functions remain unclear although some of these encode domains suggestive of toxin-like activities [6] (VA, L.M. Iyer, LA, unpublished).

We are now in a position to formulate the immunity-dormancy/suicide coupling hypothesis according to which immune systems and cell dormancy/suicide systems such as TA are functionally coupled and directly regulate each other’s functions (Figure 3). Stand-alone suicide/dormancy modules, such as typical TA systems, lack a direct, demonstrable role in immunity and might even decrease the fitness of the host, especially when they exhibit their toxic activity in the context of plasmid addiction or restriction attack upon disruption of RM systems. However, the comparative genomics data summarized here indicate that all major antivirus immunity systems identified to date in prokaryotes are linked to proteins that are homologous to toxin subunits of typical suicide/dormancy modules and are predicted to possess toxin-like activities. These toxin-like proteins might not be mechanistically involved in the immune function but rather could act similarly to their toxin counterparts from stand-alone suicide/dormancy modules.

**Figure 4:**
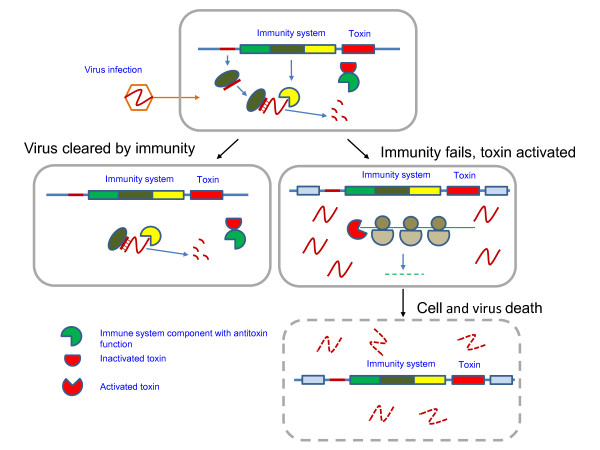
**The immunity-dormancy/suicide coupling hypothesis: Route 2 – toxin action after immunity failure.** The coupling between dormancy-suicide and immunity is illustrated by the CRISPR-Cas system as in Figure 3.

In most cases, the characterized and predicted toxin proteins in immune systems are highly variable both in terms of (potential) targets (i.e. DNA, tRNA, rRNA or mRNA) and mechanism of action (i.e. protein modification or nucleic acid degradation). However, if our prediction of the toxin-like interferase function for Cas2 is valid, the coupling between immunity and suicidal/dormancy response is an intrinsic feature of all CRISPR-Cas systems. Indeed, such coupling might be critical for the effectiveness of the adaptive CRISPR-Cas systems. Given that antivirus defense by these adaptive immunity systems depends on preliminary infection to integrate virus-specific spacer DNA, a dormancy response through the action of Cas2 is likely to help the host to ‘buy time’ to prime the immunity response. Under the current hypothesis, this is the first route of involvement of dormancy/suicide modules in coupled defense whereby the dormancy-like response precedes and enables the immune response (Figure 3). The second route of immunity-suicide coupling follows an even more straightforward biological rationale: when an immunity system fails and/or the level of genotoxic stress increases through the activity of metabolic enzymes encoded by the infecting virus, the cell employs the associated toxins for drastic measures that involve abrogation of key cell processes, typically translation, leading to cell death or dormancy (Figure 4).

Immunity systems do not encode typical antitoxins. Therefore (and by analogy with the PrrC model), we hypothesize that components of the immunity systems themselves (e.g. Cas1) function as reversible inhibitors of the toxic components, and that they are inactivated by genotoxic stress or viral counter-defenses neutralizing the immunity system and thus unleashing the toxin (Figures 3 and 4).

## Testing the hypothesis

Clearly, the immunity-suicide coupling hypothesis generates numerous predictions that can be tested experimentally on diverse prokaryotic defense systems. Such tests would involve biochemical experiments to detect the activity of predicted toxin-like proteins, such as Cas2, as well as computational searches for novel toxin-like proteins among products of completely uncharacterized genes within defense loci. Given that many of the toxins are highly diverged, small proteins [25], the latter approach is expected to yield numerous new findings. In addition, *in trans* interactions between immunity systems and TA modules cannot be ruled out. Beyond direct characterization of enzymatic activities, physical interactions between (predicted) toxins and components of immune systems (e.g. Cas1-Cas2) represent important targets for experimental testing of the hypothesis.

## Implications of the hypothesis

A general implication of the immunity-suicide coupling hypothesis is that infected bacterial or archaeal cells routinely make decisions on the nature of their response to virus infection: induction of a dormancy-like state to allow priming of immunity; immediate immune response; or dormancy/suicide in the face of immune system failure. Apparently, these decisions are made through sensing the course of the virus infection. Such decision-making could be highly complex and might potentially involve diverse signal transduction systems similarly to the sporulation-competence decision in *Bacillus subtilis* that has been mathematically modeled and experimentally characterized [63,64]. Mathematical modeling of the immunity-dormancy networks has the potential to reveal unexpected properties and functional regimes of prokaryotic defense systems.

## Competing interest

The authors declare no competing interest.

## Authors’ contributions

KSM and VA collected and analyzed the data; KSM, LA and EVK formulated the hypothesis and wrote the manuscript that was read and approved by all authors.

## Reviewers’ reports

Reviewer 1: Dr. Arcady Mushegian, Stowers Institute for Medical Research, Kansas City

The study by Makarova et al. notes that most of the operons encoding bacterial immunity systems (i.e., restriction-modification, DNA phosphorothioation, and CRISP-Cas) are associated with the known or predicted suicide/dormancy systems (i.e., toxin-antitoxin and abortive infection). The hypothesis is that bacteria are playing zone defense, which apparently can take two different forms: first, if immunity system fails to mount an efficient response to non-self DNA, then a suicide system is activated to kill the cell and prevent the completion of virus infection; second, if immunity system is slow to mount a response to non-self DNA, then a dormancy system may shift cell metabolism in low gear, perhaps slowing down virus reproduction and ‘buying time’ for the immunity system to catch up. Somehow, these two versions of the echeloning are not presented as two distinct responses, despite their opposite outcome, but are discussed interminglingly; perhaps it would be better to state them as the alternatives.

Authors’ response: *We agree that the two routes of dormancy*-*immunity coupling*, *one where dormancy is proposed to enable immunity and the other one where the transition to dormancy or actual suicide follows immunity failure*, *are best clearly distinguished*. *This is how we treat the subject in the revised manuscript including the Abstract and the modified* Figure 3*and new* Figure 4.

The idea, nonetheless, is of a considerable interest. It comes with many specific predictions of novel functions and domain architectures of the involved proteins. In addition to all this, it is hypothesized that Cas system itself has a built-in Kingston valve, consisting of the ubiquitous Cas2 component that acts as a suicidal toxin when released from its inhibitory interaction with Cas1, which has an antitoxin function. The main open question in my mind is whether the tight control of the zone defense of the suicidal variety is possible. Failure or slowness of the cell to

mount the immunity response has to be sensed neither too early (otherwise, futile destruction of the cell that was mounting said response just fine) nor too late (otherwise, destruction only releases already-formed virus progeny). This has to be recognized as an intrinsic difficulty; perhaps dormancy response is more flexible in this regard.

Authors’ response: *This comment is very much along the lines of the Implications section in the revised manuscript*. *As we note in this section*, *mathematical modeling of the coupled immunity*-*dormancy response is likely to reveal different regimes and perhaps provide at least tentative answers to the questions posed in this comment that could then become experimentally tractable*.

Minor comments:

“[defense island formation] occurs fortuitously due to non-adaptive clustering of horizontally transferred genes” --- perhaps horizontally transferred genes may also cluster adaptively, but not because of their molecular functions but because, for example of the advantage in avoiding the established genome neighborhoods?

Authors’ response: *indeed*, *this is conceivable as discussed previously*[6].

“Cas2 .. is a homolog of the VapD toxin of the VapD/VapX TA system [41-44]. Therefore it appears highly likely that analogously to VapD, Cas2 is an mRNA interferase…” --- two homologs have the same function; somehow, ‘analogously’ does not belong here.

Authors’ response: *perhaps*, *this was indeed awkward*, *modified*.

is it out of the realm of possibility that Cas-associated reverse transcriptases

could act on virus RNA, rare as the RNA bacteriophages are?

Authors’ response: *the data discussed in the article seem to point in a difference direction*; *yet*, *we agree*, *the involvement of the RT in the CRISPR*-*Cas response to RNA viruses is not entirely inconceivable*

Reviewer 2: Dr. Etienne Joly, Université Paul Sabatier, Toulouse

I have found this manuscript very difficult to get through, to the extent that it took me several hours to feel like I understood it sufficiently to write a review. The fact that the subject is not right up my field of expertise have certainly contributed to my difficulties, but I perceive that the main problem lies with the overall structure of the manuscript and the written style, with many very long and poorly structured sentences and paragraphs. The manuscript also contained obvious gramatical and typographical mistakes which I have already indicated to the authors directly, but which can be found highlighted in the pdf attached. Since I was not provided with a cover letter, and since the authors did not bother to format their paper as recommended in the instructions for authors, I had to read the manuscript several times to come to the conclusion that this was a hypothesis article. In fact, I identified as many as four separate hopytheses within this article (underlined in the attached pdf). In my opinion, the manuscript will be greatly improved if the authors make the effort of adopting the format requested for such articles. Until such times, I cannot see how I can make more constructive comments on scientific grounds.

Authors’ response: *We regret that the reviewer found the manuscript to be so impenetrable*. *The article was explicitly submitted as a Hypothesis*. *Given the formatting requirements*, *the revised manuscript was indeed restructured as recommended although it is our opinion that free format might better suit Hypotheses*. *Beyond this formal restructuring*, *we did make an effort to clearly demarcate the two aspects of our hypothesis rightly identified by Reviewer* #*1*. *We appreciate the reviewer taking the time to annotate the manuscript and we did implement most of the proposed minor modifications*. *Beyond that*, *however*, *because no substantive comments were made*, *there could be no substantive responses*.

Second review: This manuscript presents an interesting hypothesis whereby kin selection/altruism may play a very significant role in the way bacteria combat against viruses, by committing suicide (or dormancy) rather than letting the virus complete its infectious cycle. The main ground for this hypothesis is that many, if not most, antiviral operons contain proteins that appear more likely to play a role in causing the suicide (or dormancy) of the host than against viruses. My main comment is that I wish to congratulate the authors for the remarkable improvement of their manuscript compared to the initial version. The manuscript is now much easier to read and comprehend for someone like me who is not directly involved in this field: it is now much easier to see what was already known, what the speculations and hypotheses presented are, and to see how these could be confirmed or infirmed by the predictions made.

Authors’ response: *We greatly appreciate these comments*.

Reviewer 3: Dr. Nick Grishin, University of Texas Southwestern Medical Center, Dallas

In this short manuscript an interesting hypothesis is suggested that immunity and suicide in bacteria may be linked by location of immunity genes and suicide nuclease genes within the same operon. Moreover, due to the lack of known antitoxins encoded in such operons, immunity proteins may serve as reversible inhibitors of these nucleases that would cause cell death when immune response fails. The hypothesis is supported by the following evidence: abundance of nucleases without clear functional roles in the operons of all major bacterial immunity systems; extensive functional characterization of CRISPR-cas system failed to pinpoint the function of essential Cas2 suggesting that Cas1 enzymatic activity is sufficient; Cas2 is homologous to the VapD toxin (nuclease); deteriorated Cas2 proteins may be fused with other, possibly active nuclease domains. All these small pieces of incidental evidence and more are not likely to be fortuitous and indeed the hypothesis suggested by the authors seems very attractive and worthy of experimental exploration. However, it is also possible that some of these nucleases do have other functions in immunity and such functions have not been discovered yet. In any case, experimental study of an uncharacterized protein is best to start from a testable functional hypothesis and this work provides a highly plausible one. In addition, introduction and parts of the results section read like an excellent review and a primer in bacterial immunity for readers who are not particularly familiar with the subject. In these regards, I think that the following recent papers might be relevant to the discussion [4,27]

Authors’ response: *We appreciate these comments and cited the suggested*, *indeed highly relevant references in the revised manuscript*.
